# Real-time intraoperative 3D image intensifier-based navigation in reversed shoulder arthroplasty- analyses of image quality

**DOI:** 10.1186/s12891-019-2657-2

**Published:** 2019-05-30

**Authors:** Jan Theopold, Philipp Pieroh, Ralf Henkelmann, Georg Osterhoff, Pierre Hepp

**Affiliations:** Department of Orthopedics, Trauma and Plastic Surgery, Division of Arthroscopy, Joint Surgery and Sport Injuries, University of Leipzig, Liebigstraße 20, D-04103 Leipzig, Germany

**Keywords:** Proximal humerus fracture, 3D imaging, Patient safety, Shoulder, Computer assisted surgery, Shoulder arthroplasty, Navigation, Reversed shoulder arthroplasty, Shoulder osteoarthritis

## Abstract

**Background:**

Due to the high anatomical variability and limited visualization of the scapula, optimal screw placement for baseplate anchorage in reversed total shoulder arthroplasty (rTSA) is challenging. Image quality plays a key role regarding the decision of an appropriate implant position. However, these data a currently missing for rTSA and were investigated in the present study. Furthermore, the rate of required K-wire changes for the central peg as well as post-implantation inclination and version were assessed.

**Methods:**

In ten consecutive patients (8 female, 86 years, range 74–94) with proximal humeral fracture and indication for rTSA, an intraoperative 3D-scan of the shoulder with a 3D image intensifier (Ziehm Vision FD Vario 3D© [Ziehm Imaging GmbH, Nürnberg, Germany]) was performed after resection of the humeral head. Using the Vectorvision© Software (Brainlab AG, Feldkirchen, Germany), the virtual anatomy was compared to the visible anatomical landmarks. After implantation of the baseplate, a 3D scan was performed. All 3D scans included multiplanar reconstruction (MPR) and the cinemode to examine screw and baseplate placement. The rate of required K-wire changes was assessed. The intraoperative 3D image quality (modified visual analogue scale [VAS] and point system) was assessed before and after implantation of the glenoid component. Inclination and version were determined in post-implantation scans.

**Results:**

The virtually presented anatomical landmarks always correlated to the anatomical visible points indicating an good virtual accuracy. The central K-wire position was corrected in three cases due to a deviation from the face plane technique position. The VAS was higher for the pre-implantation MPR (6.7, range 5–8) compared to the post-implantation acquired MPR (5.1, range 4–6; *p* = 0.0002). The point system showed a reduced quality in all subcategories, especially regarding the grading of the articular surfaces. The preoperative (7.9, range 6–9) and post-implantation (7.9, range 6–9) cinemode displayed no significant differences (*p* = 0.6).

**Conclusion:**

The present study underlines the need for the improvement of 3D image intensifiers algorithms to reduce artifact associated impaired image quality to enhance the benefit of real-time intraoperative 3D scans and navigation.

## Background

The glenoid component stability predicts stability of total shoulder arthroplasty [1–3] and strongly depends on exact implant positioning [4]. Due to the surrounding soft tissue and the complex geometry of the glenoid, the implantation of the baseplate is technically challenging and accurate implant placement may fail [5–8]. Even with standardized methods for intraoperative baseplate orientation, like the ‘face plane’ and the ‘neutralization’ technique, the accuracy of final implant positioning remains unsatisfactory [8–11]. Computer based analyses revealed that the central peg should optimally be positioned within the normal glenoid vault [11]. Due to the opportunity to plan reversed total shoulder arthroplasty (rTSA) from computed tomography (CT) scans, the use of patient-specific implants (PSI) increased related to their suspected potentially higher accuracy [12–15]. However, the time from the first clinical presentation of the patient to the production of a PSI limits the application of such implants or patient specific guides, especially in trauma surgery. In contrast, intraoperative real- time 3D image intensifier based reconstructions combined with navigation is known to ensure accurate implant positioning and reducing complications using intraoperatively acquired images [16, 17]. Thus, navigated implantation of shoulder arthroplasty has an increasing role [4, 11, 18, 19] and might improve positioning of the glenoid component [4, 18, 20, 21]. Although a few cadaver studies suggest a benefit of intraoperative 3D imaging combined with navigation in rTSA [22, 23], clinical studies are still rare. Especially, data concerning the image quality of the real-time obtained 3D scans pre- and post-implantation are missing. Such data were previously obtained for different 3D image intensifiers determining the overall clinical applicability using a modified visual analogous scale (VAS) and a point system [24]. Probably, these data may highlight the benefit of real-time intraoperative 3D image intensifier-based navigation. We assumed that the pre-implantation scans will have a higher image quality and that the implantation of the baseplate will lead to a reduction of the quality in all subcategories, especially regarding artifacts. In addition, the quality of multiplanar reconstructions (MPR) and the cinemode were compared. Secondarily, we hypothesized that the use of 3D scan-based navigation will reliably detect visible anatomical landmarks, decrease the rate of K-wire repositioning of the central peg and yield good results in terms of version and inclination.

## Methods

All consecutive patients who met the inclusion and exclusion criteria (Table 1) and treated by rTSA for an acute proximal humerus fracture at our university Level 1 trauma center were included (*n* = 10; 8 female and 2 male; Table 2). After the exclusion of concomitant neurovascular injuries, radiographs (anterior-posterior and axial) of the shoulder were obtained. The fractures were classified according to Codman [25] and the Neer criteria [26] (Table 2). rTSA was performed in 10 cases to treat proximal humeral fractures (*n* = 8 four-part, *n* = 2 three-part dislocation fracture type VI according to Neer). The indication for surgery was made in consensus by two specialized shoulder surgeons. The present study was performed retrospectively, patients were treated between November 2011 to February 2013.

**Table 1:** Inclusion and exclusion criteria

Inclusion	Exclusion
Age ≥ 18 years	Pregnancy
Non-reconstructible proximal humeral fracture	Accompanied neurovascular injuries
Signed informed consent

**Table 2:** Patient demographics including fracture classification according to Neer, age, body mass index (BMI), time to surgery, amount of comorbidities, surgery time

Number	Classification	Age [years]	BMI [kg/m^2^]	Time to surgery [days]	Amount of comorbidities[n]	Surgery Time [minutes]
	*Distribution, n*	*Mean (range)*	*Mean (range)*	*Mean (range)*	*Mean (range)*	*Mean (range)*
Σ = 10	*n* = 8 four part *n* = 2 three part	85.6 (74–94)	27.5 (19.8–34.7)	7 (1–40)	4 (1–9)	126 (104–159)
1	four-part	94	19.8	5	3	104
2	three-part dislocation fracture type VI	83	26	40	9	117
3	four-part	74	25	1	4	159
4	three-part dislocation fracture type VI	85	26.9	7	8	114
5	four-part	84	29.3	4	1	151
6	four-part	85	34.7	1	7	143
7	four-part	87	34.7	5	2	119
8	four-part	84	25.9	1	2	111
9	four-part	89	25.6	1	6	117
10	four-part	91	27.6	1	2	128

### Intra-operative set-up and 3D fluoroscopy

**Fig. 1:**
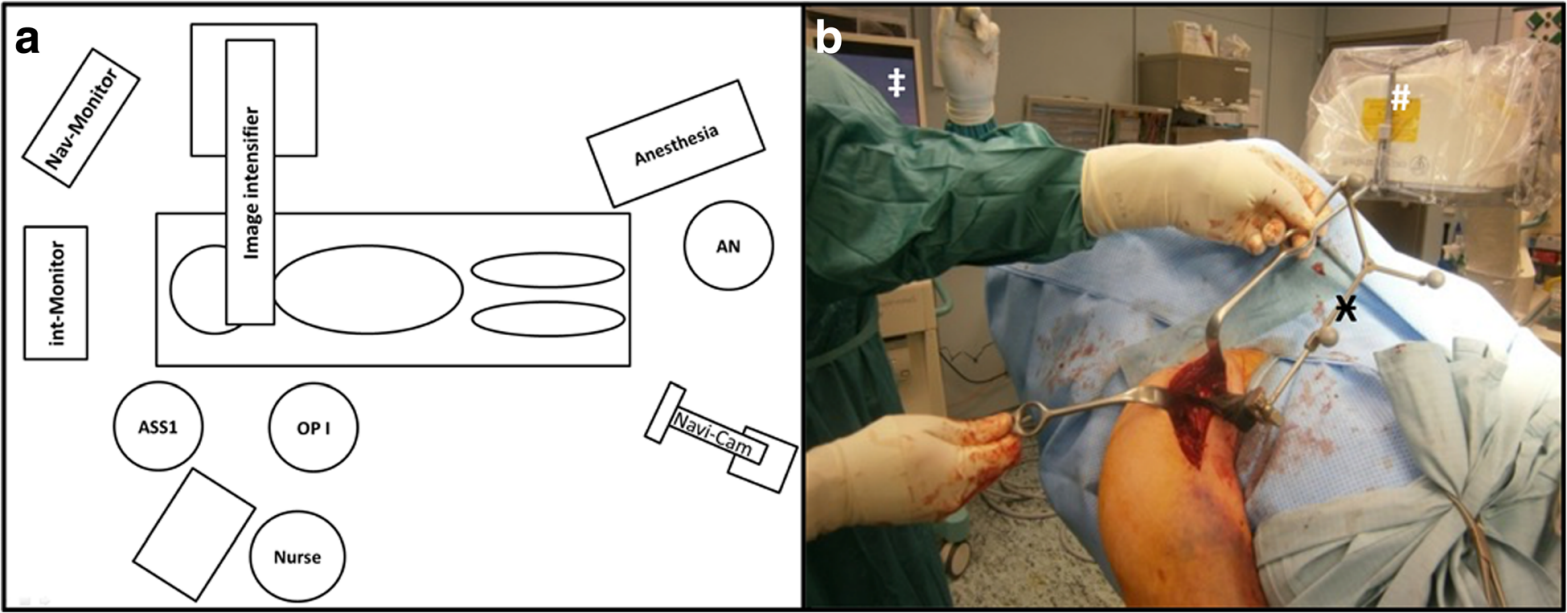
Intraoperative setup (**a**) Schematic illustration of the positions of the OR team during surgery (OPI = surgeon; ASS1 = first assistant; AN = anaesthesist) (**b**) Intraoperative situs with navigation setup from the surgeon’s perspective. The image intensifier is positioned from the opposite side to increase workspace. Ӿ Navigation clamp made of carbon with the 3 markers and attached to the coracoid. ‡ The monitor of the image intensifier is optimally visible to the surgeon at the head end of the patient

The patient is placed in a modified beach-chair position on an operation table with a carbon-fiber extension plate attached to the cranial end (Maquet, Rastatt, Germany). The anesthesia set-up is positioned at the feet. Due to this modification the true anterior-posterior and axillary views of the proximal humerus can be easily obtained using a C-arm image intensifier. The 3D image intensifier, Ziehm Vision FD Vario 3D©, Software version: 5.6.3 (Ziehm Imaging GmbH, Nürnberg, Germany) is positioned on the opposite side and the display is placed close to the head (Fig. 1).

All surgeries where performed by one experienced shoulder surgeon using the delto-pectoral approach*.* After preparation the humeral head was resected.

**Fig. 2:**
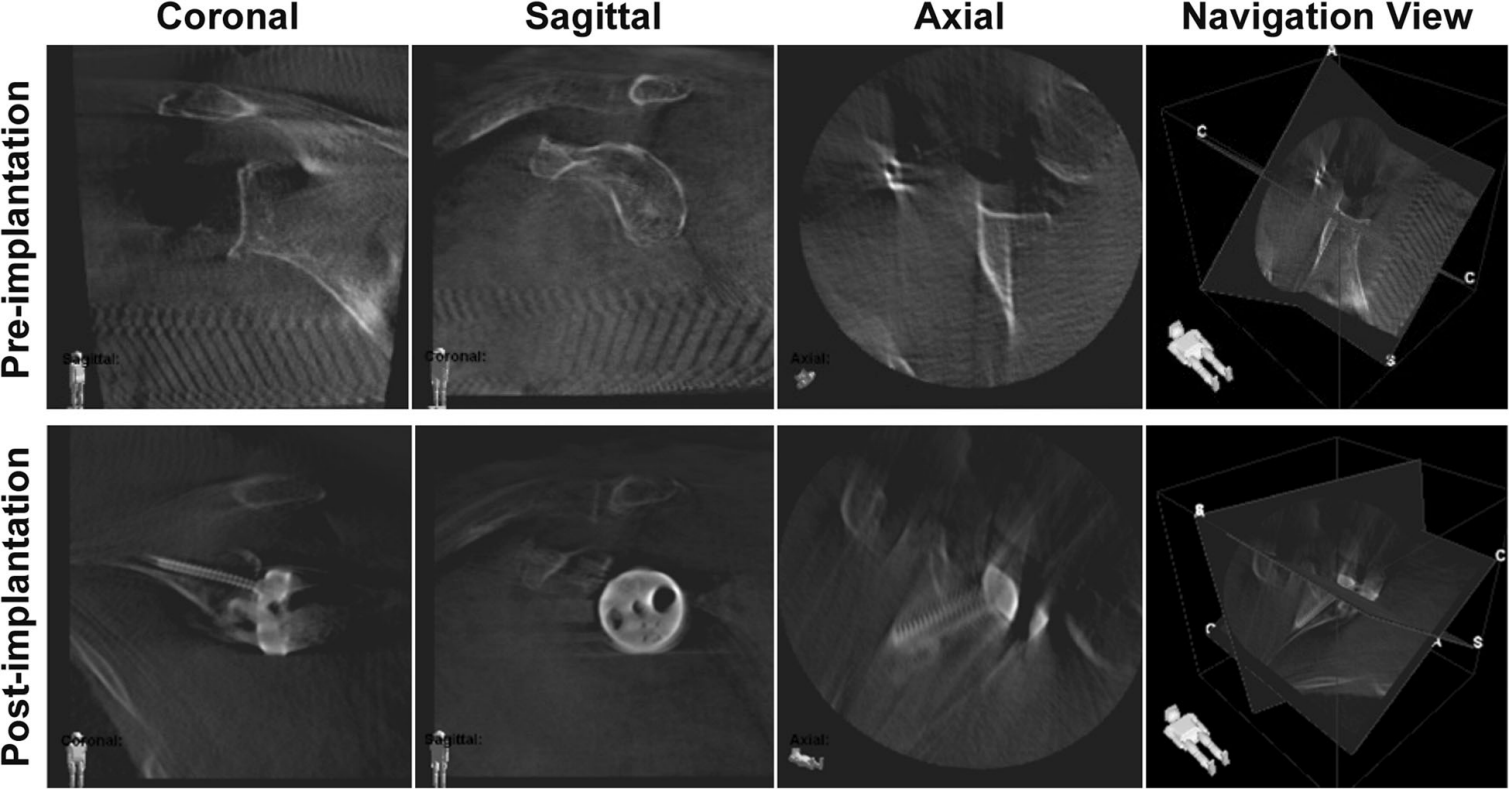
Image intensifier obtained 3D scans including multiplanar reconstructions with the navigation view of the system pre- and post-implantation of the baseplate

**Fig. 3:**
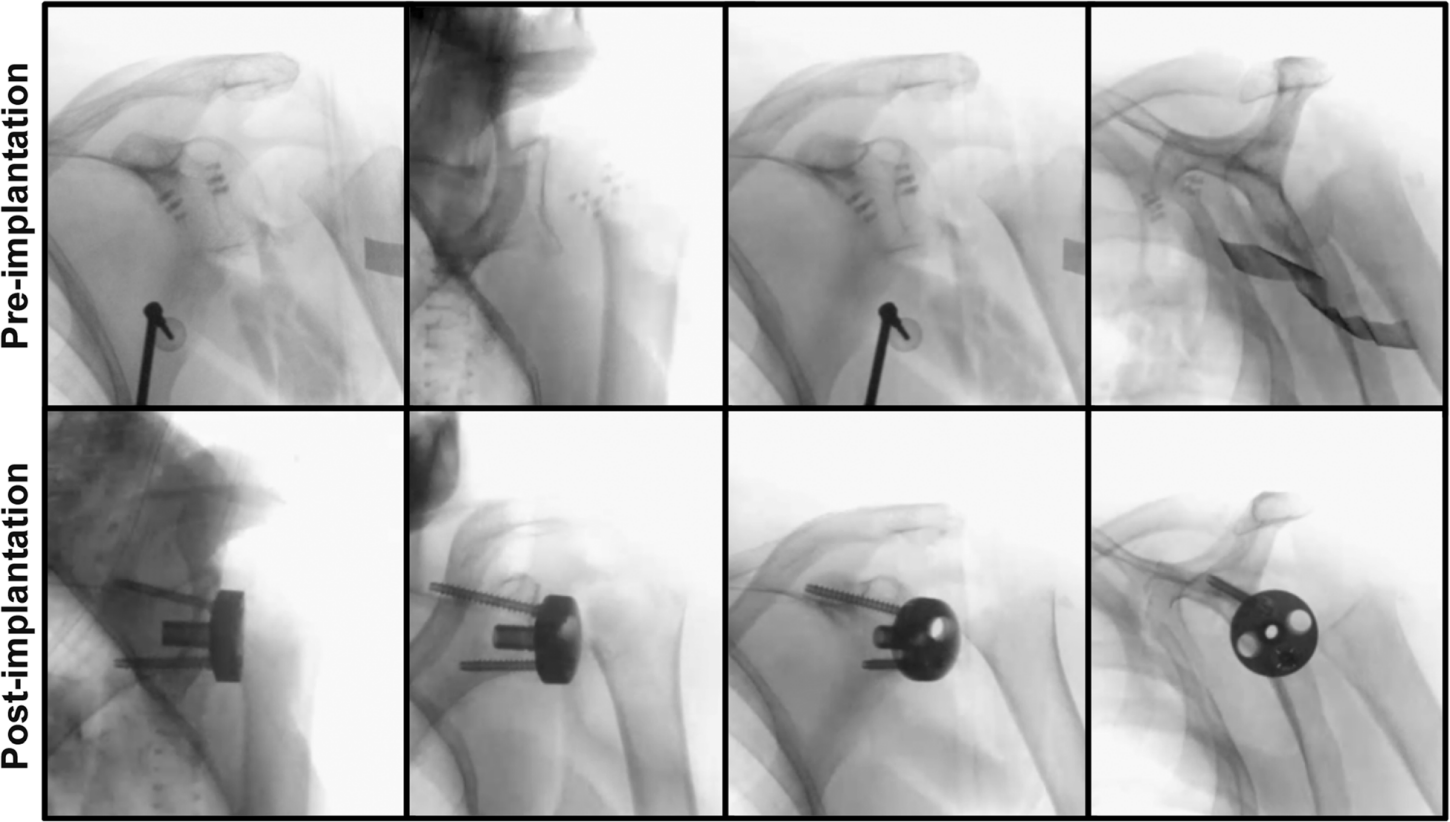
Representative isocentric image series (cinemode) pre- and post-implantation of the baseplate. Exemplary cinemode of a 85-year-old patient with a four-part fracture and metaphyseal comminution

A carbon-clamp with three tracking balls was fixed to the coracoid process as reference (Fig. 1, Ӿ). A pre-implantation scan was obtained with the scan center position at the central point of the glenoid. The scan consisted of 110 single images with a radius of 136° and took 110 s. The obtained 3D scan with multiplanar reconstructions (MPR, Fig. 2) and an isocentrically acquired image series (cinemode, Fig. 3) were controlled before navigation.

### Navigation

**Fig. 4:**
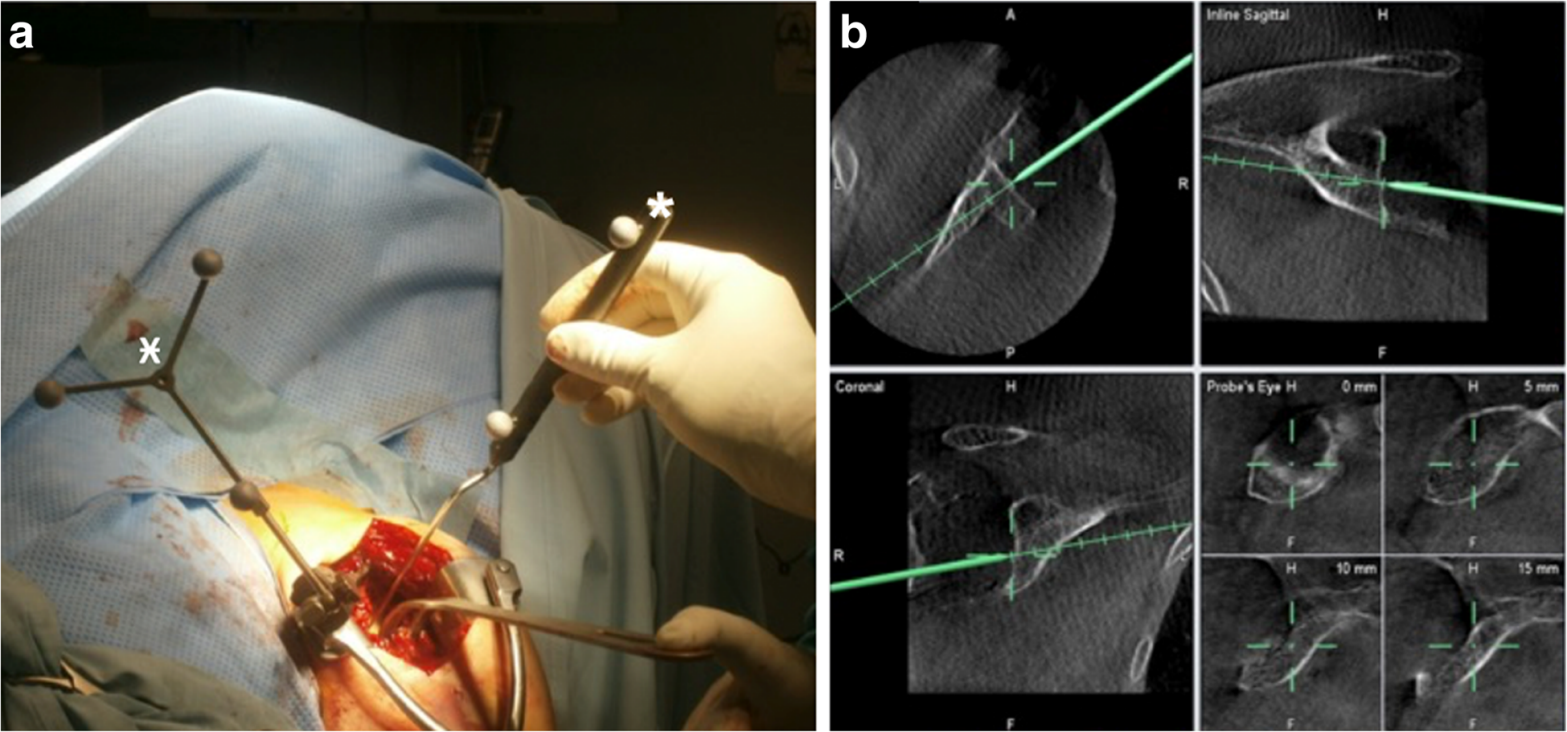
Intraoperative assessment of virtual accuracy. The reference clamp is positioned on the coracoid process (Ӿ). Following the pre-implantation 3D scan and before drilling the virtual accuracy was examined. **a** Intraoperatively, the surgeon sets the pointer (*) to previous defined anatomical landmarks (coracoid, cranial glenoid, inferior glenoid). **b** The pointer marked anatomical landmarks were controlled in the 3D scan displayed on the navigation screen in the different views (inline axial, inline sagittal and coronal). Virtual accuracy was graded excellent in case of no mismatch. A mismatch is defined as the difference between the virtually displayed and anatomical visible landmark

Raw data from the 3D image intensifier were transferred as DICOM data to the VectorVision© navigation system, Software version: Spine+Trauma 3D 2.6.0.792 (Brainlab AG, Feldkirchen, Germany). In the navigation system, the 3D image intensifier data was used instead of a CT scan. After creating a 3D image, the data were verified comparing the virtually displayed structures to the visible anatomical landmarks. This control was performed at three defined points (coracoid, cranial glenoid, inferior glenoid; virtual accuracy) (Fig. 4).

The surgical instruments were calibrated using a 1.8 mm navigable drill sleeve (Fa. Brainlab, Feldkirchen, Germany). Under navigation control the K-wire was positioned in the center of the glenoid vault. Subsequently, the baseplate (Delta extend; Fa. DePuy Orthopaedics, Leeds, England) could be implanted according to the manufacturer’s instructions. The central K-wire was changed if a mismatch to the “gold standard”, namely the face plane technique, was detected. This criterion was introduced to ensure a similar safety for the participating patients as during conventional rTSA.

### Investigated parameters

The duration of surgery was recorded and compared to 30 arbitrarily selected patients with a comparable age and fracture morphology who underwent rTSA to treat a proximal humeral fracture. The virtual accuracy between the visible anatomical reference points (the coracoid, the cranial glenoid rim, and the inferior glenoid rim) to the virtually presented anatomical landmarks was determined (Fig. 4). A mismatch was defined as relevant if the estimated difference between anatomical and virtual landmark was more than 2 mm. Here, only a match or mismatches were permissible answers. The rate of required K-wire repositioning was assessed.

The inclination and version of the baseplate in the post-implantation scans were measured accordingly to Stübig et al. using the OsiriX DICOM shareware viewer (OsiriX.com) [22]. Glenoidal version was determined, detecting the first cut below the coracoid process and measuring the position of the K-wire to the scapular axis [4]. The inclination or component tilt was examined in the sagittal plane relative to the frontal plane of the scapula [4]. The image quality of the pre- and post-implantation (after implantation of the baseplate) 3D scans including MPR (Fig. 2) was graded as previously described [24]. Here, a modified visual analogous scale (VAS) and a point system grading the image quality and clinical applicability were applied [24]. A score of 10 in the VAS represents an excellent picture whereas a score 0 determined the picture as not usable. The VAS was calculated for MPR (Fig. 2) and 3D image intensifier calculated isocentric cine loops based on the 2D fluoroscopy images (*n* = 110) obtained during the 3D scan (“cinemode“; Fig. 3). The cine loops are movies showing the complete sequence of obtained 2D fluoroscopic images obtained during one 3D scan with a fixed scan center. The criteria of the point system are summarized in Table 3 and were determined for MPR.

**Table 3:** Point system according to Stübig et al. [24]. Subjective Image Quality Total (SIQ), trabecular structure (TS)

Points	SIQ	Delineation of Cortical Bone	Delineation of Cancellous Bone	Delineation of Articular Surfaces	Artifacts	Clinical Assessment Total
1	Excellent	Excellent	TS perfectly visible	Excellent	No relevant artifacts	Very good evaluation, no open questions
2	Good	Good	TS clearly visible	Good	Few artifacts, barely disturbing	Good evaluation despite minor quality defects
3	Acceptable	Acceptable	TS moderately visible	Acceptable	Moderate artifacts, slightly disturbing	Evaluation generally possible with some open questions
4	Somewhat reduced	Barely visible, blurred edges	TS barely visible	Barely visible, blurred edges	Disturbing, evaluation rather limited	Limited evaluation, control scan recommended
5	Reduced	Completely blurred, no delineation	TS not visible	Completely blurred, no delineation	Very disturbing, evaluation impossible	No evaluation of query, CT recommended

Results are reported as mean and range. The statistical analysis was done via SPSS (IBM, Version 24). The Mann-Whitney test was used. The level of significance was set to *p* < 0.05.

## Results

No coracoid fracture occurred related to the fixation of the carbon clamp. The mean time for surgery was 126 min (range, 104–159 min) for rTSA with navigation compared to 80 min (range, 40–131 min) without navigation. rTSA without navigation yielded a significant shorter surgery time (*p* < 0.05).

The correlation of the visible anatomical points to the structures virtually presented during navigation was good for all used reference points (the coracoid, the cranial glenoid rim, and the inferior glenoid rim). No mismatch was detected representing an excellent virtual accuracy.

In three cases the central K-wire for the peg position was corrected due to a visually subjective mismatch to the face plane technique [9] but not related to an unfavorable course of the K-wire yielded by the 3D scan.

The MPR generated by the 3D image intensifier pre-implantation yielded a higher VAS (6.7, range 5–8) compared to the post-implantation acquired MPR (5.1, range 4–6; *p* = 0.0002; Table 4) (Fig. 2). Post-implantation, predominantly the differentiation between cancellous and cortical bone in the MPR was impaired due to metal artifacts and given as reason for lower VAS. The comparison of the rating of the cinemode pre-implantation (7.9, range 6–9) and post-implantation (7.9, range 6–9) displayed almost similar results (*p* = 0.6) (Fig. 3). In the point system according to Stübig et al. within all categories, a reduction of quality was found in post-implantation scans (Table 5). This quality reduction was pronounced regarding the delineation of articular surfaces. The mean inclination was – 3.2° (range, − 7.4-3.4°) and mean version was − 1.6° (range, − 14.2-5.4°; Table 6) detected in post-implantation scans.

**Table 4:** VAS scores for each patient pre- and post-implantation and for MPR and the cinemode

Number	VAS MPR		VAS Cinemode	
	*pre-implantation*	*post-implantation*	*pre-implantation*	*post-implantation*
Σ = 10	6.7 (5–8)	5.1 (4–6)	7.9 (6–9)	7.9 (6–9)
1	6	5	6	6
2	7	4	7	7
3	8	4	9	9
4	6	6	8	8
5	7	5	9	9
6	8	6	9	9
7	6	6	6	6
8	8	5	7	8
9	5	5	9	9
10	6	5	9	8

**Table 5:** Results of the point system for the MPR pre- (PreI) and post-implantation (PosI) according to Stübig et al. for each patient [24]

Number	SIQ		Delineation of Cortical Bone		Delineation of Cancellous Bone		Delineation of Articular Surfaces		Artifacts		Clinical Assessment Total	
	*PreI*	*PosI*	*PreI*	*PosI*	*PreI*	*PosI*	*PreI*	*PosI*	*PreI*	*PosI*	*PreI*	*PosI*
Σ = 10	2.2 (1–3)	3.5 (3–4)	1.2 (1–2)	3.3 (2–4)	2.1 (2–3)	3.7 (3–4)	1.4 (1–2)	5 (5–5)	2.5 (2–3)	4 (4–4)	2.1 (2–3)	3.3 (3–4)
1	2	3	1	2	2	3	1	5	3	4	2	3
2	2	3	1	3	2	4	2	5	2	4	2	3
3	3	4	1	4	2	4	1	5	3	4	2	3
4	3	4	2	4	3	4	2	5	3	4	2	4
5	2	3	1	3	2	3	1	5	2	4	2	3
6	3	3	2	3	2	4	2	5	3	4	3	3
7	2	4	1	4	2	4	2	5	3	4	2	4
8	2	4	1	3	2	4	1	5	2	4	2	3
9	1	3	1	3	2	3	1	5	2	4	2	4
10	2	4	1	4	2	4	1	5	2	4	2	3

**Table 6:** Inclination and version of the base plate’s central screw in the post-implantation scans

Number	Inclination [°]	Version [°]
	*Mean (range)*	*Mean (range)*
Σ = 10	−3.2 (−7.4–3.4)	−1.6 (−14.2–5.4)
1	−7,4	5,4
2	−2,3	−14,2
3	−2,5	−3,3
4	3,4	4,4
5	−6,7	4,8
6	−5,3	4,6
7	2,6	−8,2
8	−4,3	−8,3
9	−6,2	−4,3
10	−3,2	3,3

## Discussion

The present study supports our suggestion that post-implantation 3D scans present a lower image quality compared to pre-implantation scans. However, the VAS of the cinemode yielded no significant alterations. Although we assumed no K-wire corrections, in three cases a revision of the central K-wire was required related to a mismatch to the face plane technique. The navigation system correctly showed the pointer marked and directly visible anatomical landmarks (virtual accuracy) and yielded good results in terms of inclination and version. The present study showed that navigation with the patient in the beach chair position is possible. Though one might suspect the coracoid process vulnerable to fracture during tracker placement, we did not observe a coracoid fracture within the present study.

In contrast, the O-arm based navigation - as possible alternative [27]- requires the patient in lateral position or a change of the initial position possibly increasing the duration of the surgery [27]. Recent studies reported on a significant improvement in the glenoid component positioning using navigation [4, 18, 20]. However, all these studies used CT-based navigation or planned the surgery using a preoperative CT scan. Thus, real-time imaging following fracture reduction or following prosthesis implantation is not possible and the intraoperative control is limited [8, 22]. In contrast to CT-based navigation, 3D image intensifier based navigation delivers images intraoperatively and imaging after specific surgical steps is possible. However, this comes along with a lower image resolution. In a feasibility study using cadavers, 3D C-arm navigation for baseplate placement in rTSA, improved accuracy in positioning the glenoid baseplate was reported [22]. Nonetheless, data on the image quality are missed and may underline the benefit of 3D image intensifier-based navigation. Previously, the here used VAS score and point system were used to compare different image intensifier enabling 3D scanning [24]. With this system the clinical applicability was assessed. But so far, currently data comparing the pre- and post-implantation status are lacking. However, this data may help to improve the technology of 3D image intensifier to improve the intraoperative control in rTSA.

The image quality of mobile 3D devices is limited, in comparison to conventional CT scanners [28, 29]. Nevertheless, an evaluation of the bone morphology and implant position is possible [28, 30]. In the present study, an appropriate picture quality was determined. The preoperative scan showed a low number of artifacts and an appropriate opportunity to grade bony defects intraoperatively. The post-implantation scans showed an impaired quality compared to the preoperative scans most plausible due to the strong artifacts through the screws and baseplate.

As reported previously the measurements of a navigation system were comparable to radiographic or CT measurements regarding humeral and glenoid inclination and retroversion indicating a high accuracy of virtually and anatomical visible landmarks [8]. In the present study, no mismatch between virtually displayed anatomical structures on the navigation screen and in-situ visible anatomical landmarks was determined. Nonetheless, three K- wires were changed although an optimal positioning was yielded by the navigation system. These changes were performed solely based on subjective surgeon dependent criteria e.g. a deviation to the face plane technique or a lack of confidence to the navigation system. No objective criteria indicated a malpositioning. “Three replacements were performed to ensure the patients safety using the criterion applied in conventional rTSA, namely the face plane technique. Probably, the course of the central K-wire in those three cases was not completely false but due to degenerative changes of the glenoid morphology [31]. These changes might contribute to a deviation of the glenoid centerline and face plane technique and have led to the exchange. The post-implantation measurements of version and inclination yielded comparable results as found previously in a cadaver study [22].

The present study is limited by the small sample size and a missing control. Thus, the beneficial effects of a 3D image intensifier should not be overestimated. Larger clinical studies including a control group and specific anatomical measurements are needed to determine the advantages of 3D image intensifier-based navigation in rTSA. The present study aimed to present the first experience with intraoperative real- time navigation in rTSA especially regarding image quality.

A 3D scan was performed after the implantation of the baseplate. In terms of implant position and reconstruction this technique is comparable to a CT scan. Thus, an additional postoperative CT scan was not performed to avoid further radiation for the patient [28, 32].” Also, follow-up data regarding revision arthroplasty, outcome and implant stability should be included and are required.

## Conclusion

Intraoperative 3D image intensifier-based navigation in rTSA allows for accurate virtual real-time visualization of the anatomy supporting intraoperative decision-making, drilling control and serves as a control after glenoid positioning. The image quality seems currently inferior to conventional CT but appropriate to assess implant positioning. However, the detection of cortical and cancellous delineation as well as to grade articular surfaces is impeded. The present study underlines the need for the improvement of 3D image intensifiers algorithms to reduce artifact associated with impaired image quality. To the authors knowledge this is the first report presenting the combination of 3D image intensifier-based 3D scans and navigation in rTSA in a clinical setup and compares image quality pre- and post-implantation of the baseplate [22].

## Data Availability

The datasets used and analyzed during the current study available from the corresponding author on reasonable request.

## Abbreviations

2D: Two- Dimensional
3D: Three- Dimensional
CT: Computed tomography
DICOM: Digital Imaging and Communications in Medicine
K-Wire: Kirschner wire
MPR: multiplanar reconstructions
PSI: patient-specific implants
rTSA: reversed total shoulder arthroplasty
VAS: visual analogous scale
